# MSGU-Net: a lightweight multi-scale ghost U-Net for image segmentation

**DOI:** 10.3389/fnbot.2024.1480055

**Published:** 2025-01-06

**Authors:** Hua Cheng, Yang Zhang, Huangxin Xu, Dingliang Li, Zejian Zhong, Yinchuan Zhao, Zhuo Yan

**Affiliations:** ^1^Chengdu Civil Aviation Information Technology Co., Ltd, Chengdu, China; ^2^The College of Artificial Intelligence, Shenyang Aerospace University, Shenyang, China; ^3^Shenzhen Institute of Advanced Technology, Chinese Academy of Sciences, Shenzhen, China

**Keywords:** image segmentation, U-Net, lightweight neural network, SPP-Inception, multi-scale

## Abstract

U-Net and its variants have been widely used in the field of image segmentation. In this paper, a lightweight multi-scale Ghost U-Net (MSGU-Net) network architecture is proposed. This can efficiently and quickly process image segmentation tasks while generating high-quality object masks for each object. The pyramid structure (SPP-Inception) module and ghost module are seamlessly integrated in a lightweight manner. Equipped with an efficient local attention (ELA) mechanism and an attention gate mechanism, they are designed to accurately identify the region of interest (ROI). The SPP-Inception module and ghost module work in tandem to effectively merge multi-scale information derived from low-level features, high-level features, and decoder masks at each stage. Comparative experiments were conducted between the proposed MSGU-Net and state-of-the-art networks on the ISIC2017 and ISIC2018 datasets. In short, compared to the baseline U-Net, our model achieves superior segmentation performance while reducing parameter and computation costs by 96.08 and 92.59%, respectively. Moreover, MSGU-Net can serve as a lightweight deep neural network suitable for deployment across a range of intelligent devices and mobile platforms, offering considerable potential for widespread adoption.

## 1 Introduction

In recent years, deep neural networks have been widely used in different fields (Chollet, 2017; Wu et al., 2022; Wu et al., 2024), and their application in image segmentation has achieved good results (Hattie et al., 2024; Pang et al., 2024). This benefits from the advantages of deep learning technology in image processing, including the fact that this technology can automatically learn the characteristics and rules of the target (Yu et al., 2020), does not need to manually design features and rules, can process large-scale data, can improve segmentation accuracy and efficiency, etc. In 2014, Long et al. (2015) proposed a deep learning network, named FCN (fully-connected network), for image semantic segmentation. This network is based on a convolutional neural network (CNN), but unlike the traditional CNN, FCN replaces the fully connected layer with a convolutional layer, so that the network can accept input images of any size and output the pixel-level prediction results of the corresponding size. The performance of FCN was outstanding in image segmentation tasks at that time, and it became one of the classical models in the field of image segmentation. In 2015, researchers were inspired by the FCN architecture and the encoder–decoder model and proposed U-Net (Ronneberger et al., 2015), an encoder–decoder-based network that can segment an input image into multiple pixel-level regions. Each region is assigned a label, the encoder part consists of multiple convolutional layers and pooling layers to extract the features of the input image, and the decoder part consists of multiple up-sampling layers and convolutional layers to map the feature map extracted by the encoder back to the original image size and generate pixel-level segmentation results. The skip connection connects the feature maps of the encoder with the corresponding feature maps of the decoder, thus improving the accuracy of the segmentation. In 2015, Chen et al. (2014) accompanied the Google team proposed the Deeplab series. DeepLabv1 is based on VGG16, which uses atrous convolution and conditional random fields (CRFs) to cooperate with each other. It has achieved excellent performance in the field of image segmentation. In 2017, the authors proposed DeepLabv2 (Chen et al., 2017) based on ResNet101, which makes more flexible use of atrous convolution and proposes atrous spatial pyramid pooling (ASPP). Subsequently, Chen et al. (2017) proposed DeepLabv3, which combines cascading and parallel extended convolution modules. The parallel convolution modules are grouped in ASPP, and 1 × 1 convolution and batch normalization were added to ASPP. In order to further improve the performance of U-Net segmentation, Zhou et al. (2019) proposed (UNet++) (Codella et al., 2019), which added a convolutional neural network with nested and dense skip connections on the basis of U-Net to improve the performance of image segmentation. In 2020, UNet3 + was proposed by Huang et al. (2020). It uses full-scale skip connections to fuse feature maps at different scales and learns feature expressions from multi-scale aggregated feature maps through deep supervision. Combining the classification task with the segmentation task can enhance organ boundaries and reduce the over-segmentation of non-organ images, resulting in more accurate segmentation results. In addition to the above network architectures, extended networks based on the U-Net framework include EC-CaM-UNet (Xu et al., 2024a), Attention U-Net (Jensen et al., 2018; Codella et al., 2019), KiU-Net (Valanarasu et al., 2020; Valanarasu et al., 2021a), 3D U-Net (Çiçek et al., 2016), V-Net (Milletari et al., 2016), Y-Net (Mehta et al., 2018; Codella et al., 2019), etc., which are all based on the basic framework of the U-Net network and have demonstrated improved performance compared to that of the U-Net network.

Transformer is a neural network model based on a self-attention mechanism; it was originally proposed for natural language processing. In recent years, increasing studies have shown that a transformer model can also achieve good performance in the field of computer vision. Vision transformer (ViT) (Dosovitskiy et al., 2021) is a transformer-based computer vision model. ViT uses pre-training techniques similar to those in the field of natural language processing and has achieved good results in various computer vision tasks by pre-training models on large-scale unlabeled data. The promising applications of transformers were demonstrated in the field of computer vision. Recently, many transformer-based network architectures have been used for image segmentation, and their powerful global understanding ability can effectively help image segmentation. TransUNet (Chen et al., 2021) modified the ViT architecture to U-Net for 2D medical image segmentation. TransUNet is a modified version of ViT (vision transformer) architecture specifically designed for 2D medical image segmentation. It uses a U-Net-like architecture and incorporates the self-attention mechanism of ViT, which enables the model to learn local and global features from input images. Thus, the segmentation accuracy can be improved. TransFuse (Zhang et al., 2021) combines CNN with transformer in a parallel way, so that the network can effectively capture global dependencies and low-level spatial details in a shallower fashion and improve the efficiency of the network in modeling global context. Other transformer-based networks, such as MedT (Valanarasu et al., 2021b), TransBTS (Wang et al., 2021), UNETR (Hatamizadeh et al., 2022) and MedNeXt (Saikat et al., 2023), have been proposed and used in medical image segmentation tasks.

Image segmentation technology is widely used in the practical application of the combination of medicine and engineering, which is one of the key steps in clinical practice (Çiçek et al., 2016; Milletari et al., 2016; Mehta et al., 2018). At present, the mainstream research directions in the field of medical imaging include semi-supervised and self-supervised learning (such as multimodal contrastive learning; Zhang et al., 2023) and adaptive masking (Xu et al., 2024b), aiming to achieve excellent performance with fewer images. Many researchers have also achieved the improvement of image segmentation performance by integrating reinforcement learning and deep learning (Duan et al., 2022). Most of the existing work focuses on improving performance (Benčević et al., 2023; Chen et al., 2014; Chen et al., 2017; Chen et al., 2017), rather than on computational complexity and inference time. Rapid imaging helps clinicians expand service options and facilitates patient life, and such applications enable patients to obtain final computed tomography (CT) diagnoses without having to visit radiology centers. For example, medical imaging mobile applications and camera-based images from mobile phones are used to detect and diagnose skin conditions, and magnetic resonance imaging machines have also been developed for bedside manipulation and rapid analysis. However, most of the current deep learning based network frameworks have large computational overhead and many parameters (Soumya and Sabu, 2023; Yang et al., 2024; Zhang et al., 2021). They are difficult to embed into mobile devices.

In order to promote the effective solution of the above problems, a new network architecture named MSGU-Net is proposed in this work for application in image segmentation. This is an image segmentation network based on the Ghost (Tang et al., 2022) module. The encoder–decoder stage of MSGU-Net is designed as a Ghost module. Through the strategies of feature enhancement, computational efficiency improvement, parameter sharing, and network capacity control, the performance and computational efficiency of the network are improved. In the encoder, MSGU-Net optimizes the inception (Chollet, 2017; Ioffe and Szegedy, 2015; Szegedy et al., 2015; Szegedy et al., 2016; Szegedy et al., 2017) module and captures features of different scales by applying convolution kernels of different sizes to the input feature map. This design enables the network to learn the hierarchical structure of the image more effectively. After combining the SPP-Inception module and Ghost module, the ELA (Xu and Wan, 2024) mechanism is added to improve the network’s focus on the area of interest, provide long-distance dependencies, and improve the performance of the model. In the decoder, MSGU-Net adds an attention gate (Jensen et al., 2018) so that it can effectively extract important local features during the up-sampling process. The lightweight U-Net designed in this paper enables the network to effectively serve all kinds of intelligent mobile terminal devices. At the same time, it has less computational overhead, fewer parameters, faster inference time, and can maintain good performance. To summarize, the main contributions of this paper are as follows:

1. The proposed MSGU-Net incorporates an SPP-Inception module for feature extraction, effectively enhancing multi-scale feature fusion capabilities. Additionally, the convolutional part is designed as a Ghost module, providing a lightweight processing of the network.
2. The encoder stage is designed with an ELA (efficient local attention) mechanism, which strengthens the long-distance dependencies between pixels.
3. The decoder stage utilizes an attention gate, which autonomously learns the regions of interest and suppresses the background areas, thereby enhancing the delineation of segmentation boundaries.

## 2 Materials and methods

In this section, the overall network architecture is demonstrated along with the specific principles and functions of each module. Additionally, an analysis is provided to explain why the modules were combined in this manner.

### 2.1 Data

The ISIC2017 and ISIC2018 datasets are International Skin Imaging Collaborative Challenge datasets (ISIC2017; Berseth, 2017) and (ISIC2018; Codella et al., 2019), two publicly available skin lesion segmentation datasets containing 2,150 and 2,694 dermoscopic images labeled with segmentation membranes, respectively. In previous work, the dataset was split into a 7:3 ratio for training and test sets. Specifically, for the ISIC2017 dataset, the training set consists of 1,500 constituent images and the test set consists of 650 images. For the ISIC2018 dataset the training set contains 1886 images while the test set contains 808 images.

### 2.2 MSGU-Net network architecture

**Figure 1:**
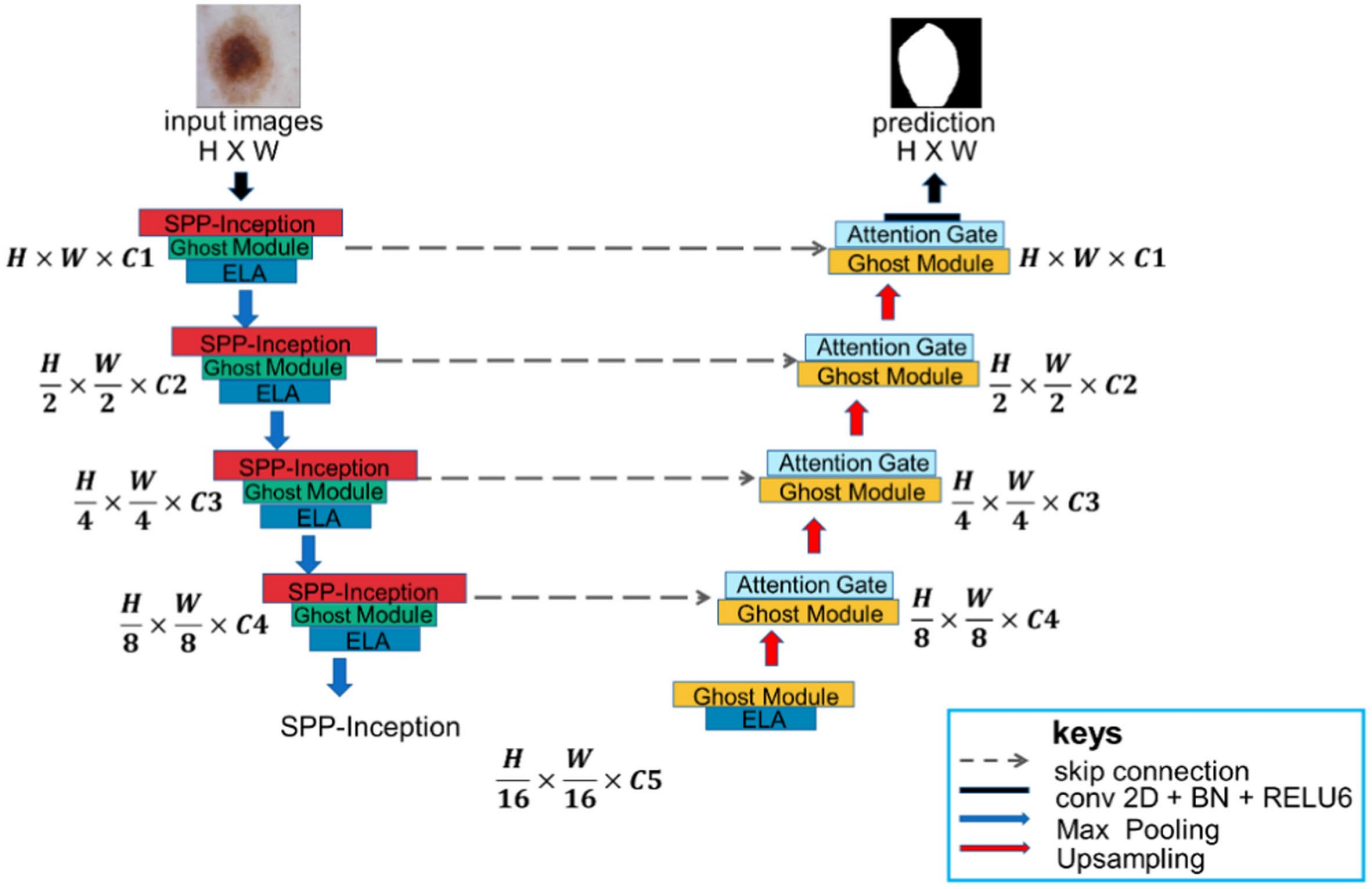
Overview of MSGU-Net network architecture.

MSGU-Net, as shown in Figure 1, is an encoder-decoder architecture that employs a U-shaped design similar to U-Net, with skip connections, but it modifies the construction of each stage. In each stage of MSGU-Net down-sampling, two kinds of modules were concerned. One module was the SPP-Inception, which mainly deals with multi-scale feature fusion so that the network can learn multi-scale features in the feature extraction stage. The other module was the Ghost module. In this work, the Ghost module was lightweight and played the role of extracting features. After these two modules, the ELA mechanism was added to accurately locate the ROI region, improve long-distance dependencies, and strengthen the connection between pixels. In the process of up-sampling, the Ghost module with an attention gate was uniformly used. The role of the Ghost module here was the same as that of the down-sampling, and the attention gate was mainly used to concentrate the local attention of the feature map in order to better process the information of the feature map in the up-sampling. Skip connections were also applied between encoder and decoder. The number of channels on each block is a hyperparameter, denoted as C1 to C5. When using the MSGU-Net architecture, C1 = 32, C2 = 64, C3 = 128, C4 = 256, and C5 = 512 were followed. Note that these numbers are smaller than the number of filters in the U-Net series, which helps to reduce the number of parameters and computations.

In the down-sampling process, each stage was equipped with an SPP-Inception module, Ghost module, and ELA attention mechanism. The batch normalization layer and ReLU (rectified linear unit) activation function were used in each module and attention mechanism. In the process of up-sampling, each stage was equipped with a Ghost module and attention gate. A max pooling layer was also added in the encoder with a window size of 2×2 , while in the decoder a 2×2 bilinear interpolation layer was used to up-sample the features. The main reason for using bilinear interpolation instead of transposed convolution was to adapt to the mobile app with fewer parameters.

#### 2.2.1 SPP-Inception module

The Inception module is a key component in Google’s Inception network. It captures features at different scales by applying different sizes of convolution kernels to the input feature maps. This design allows the network to learn the hierarchical structure of the image more efficiently. The traditional Inception module consists of multiple parallel convolutional layers, each of which processes input feature maps of different sizes. These convolutional layers typically include 1×1 , 3×3 , and 5×5 convolutional kernels and a 3×3 max pooling layer. After each branch is processed, the results are concatenated in the channel dimension to form the final output feature map. Although the Inception module is very effective in capturing multi-scale features, it uses many parameters, which may lead to model training difficulties, overfitting, and high consumption of computational resources.

A slight adjustment was carried out to the number of output channels during feature extraction:

- Adjustment of the number of output channels: At the end of each convolution branch, the number of output channels was divided by four. Doing so significantly reduced the number of parameters per branch while maintaining the expressive power of the model.
- Unified number of channels: In the improved module, the number of output channels of each branch ( 1×1 , 3×3 , and 5×5 and 3×3 max pooling followed by 1×1 convolution) was uniformly adjusted to one-quarter of the original design. In this way, the total number of channels after the CONCAT operation was exactly the desired number of output channels without further adjustment.
- Reduced computation: By reducing the number of output channels per branch, the improved Inception module reduces computation and memory requirements while maintaining performance.

**Figure 2:**
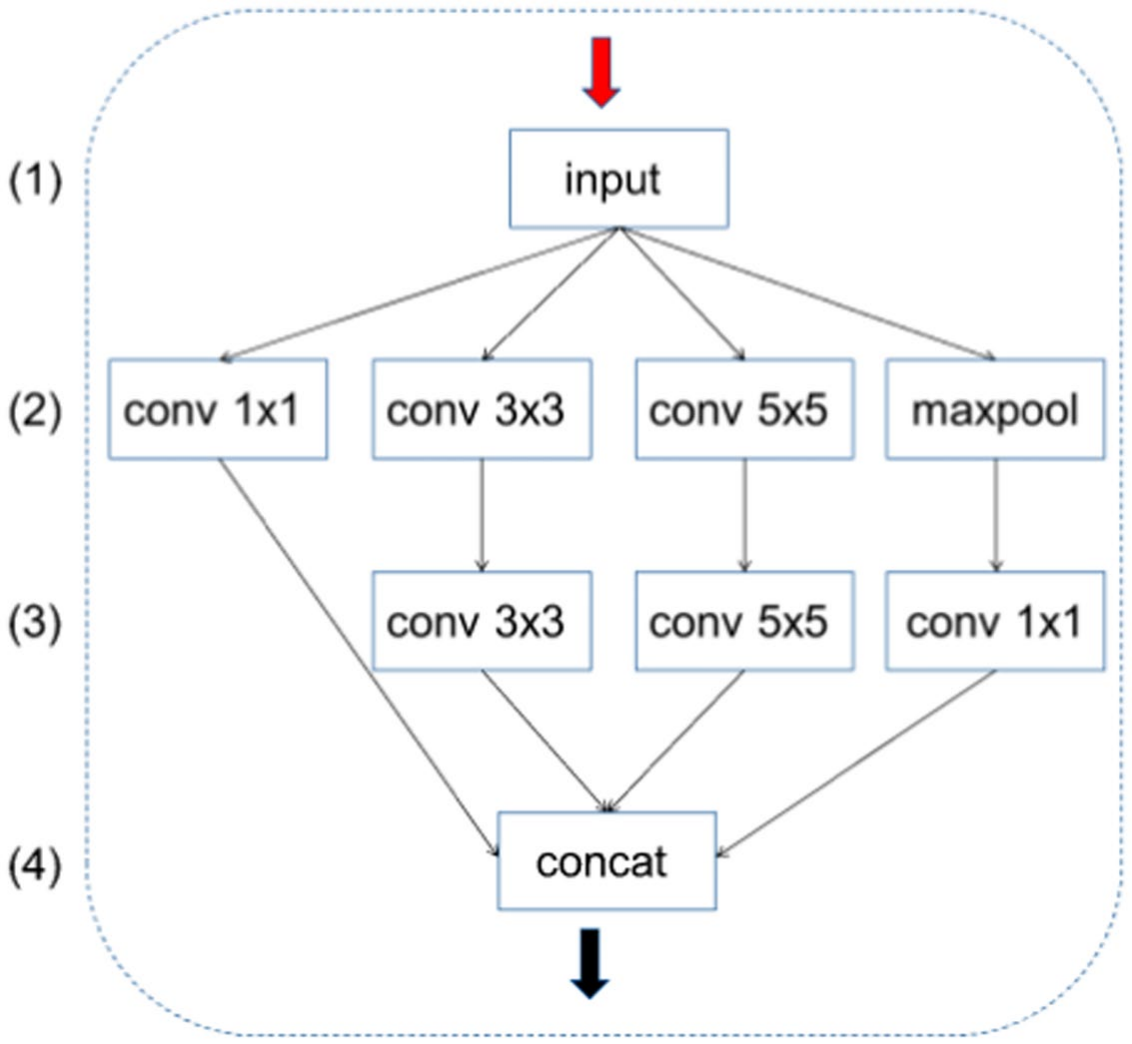
Overview diagram of SPP-Inception.

Specifically, this feature extraction is divided into four channels in parallel; therefore, the final output channel of each channel was divided by four so that the number of channels after the CONCAT operation does not need to be adjusted, and the number of parameters output by each channel is also effectively controlled. As shown in Figure 2, the improved Inception module changes the number of output channels of each branch in layer (2) to C1, C2, C3, and C4, respectively. The improved module adjusts the number of these channels to C1/4, C2/4, C3/4, C4/4, and C4/4. (3) The number of output channels of each branch in the layer is C1/4, C2/4, C3/4, and C4/4. In this way, the total number of channels after the CONCAT operation is C1/4 + C2/4 + C3/4 + C4/4 = (C1 + C2 + C3 + C4)/4, which helps to reduce overfitting and improve the generalization ability of the model. The number of channels of C1, C2, C3, and C4 is equal; the final result is exactly the number of output channels need; and there is no need to modify the number of channels.

#### 2.2.2 Ghost module

**Figure 3:**
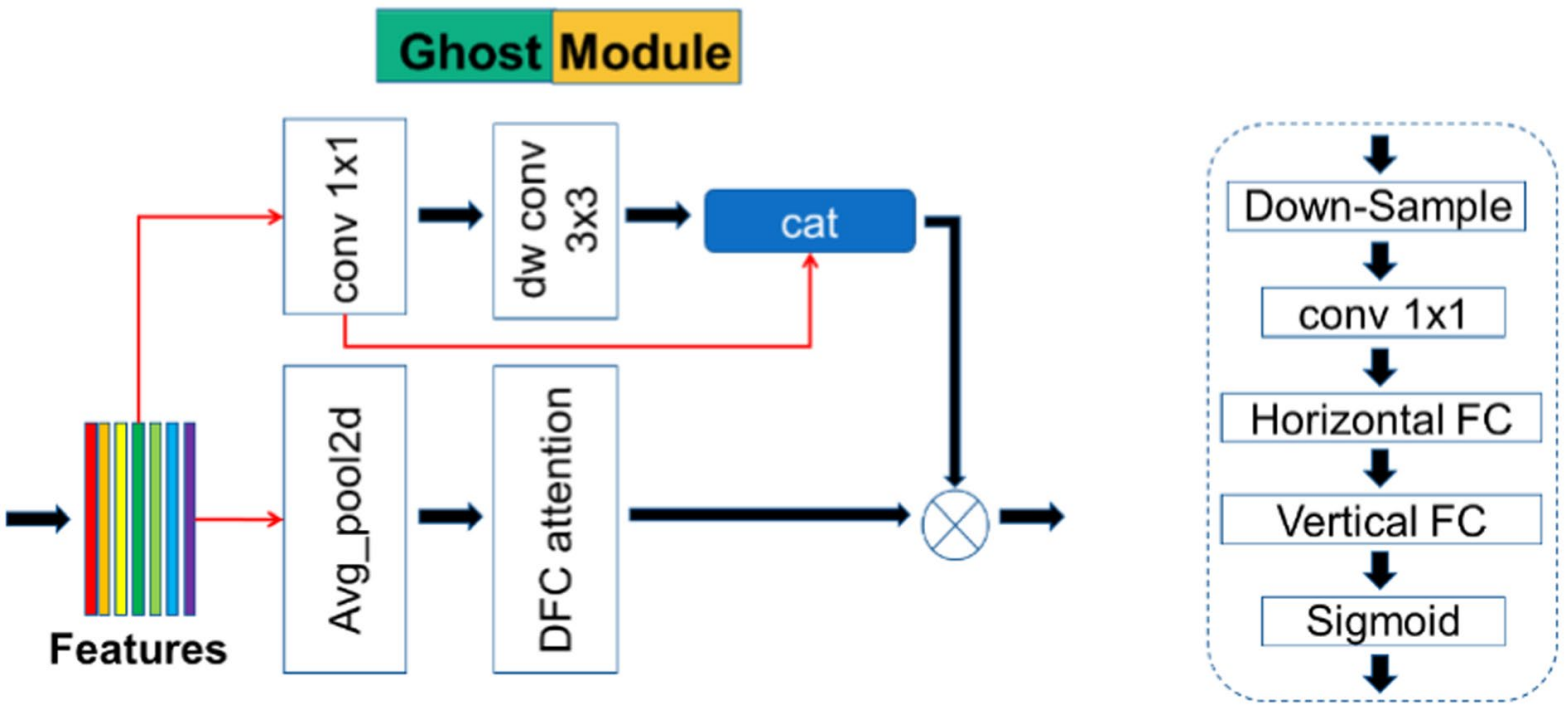
Overview diagrams of Ghost module and DFC (dynamic functional connectivity) attention.

The detailed structure of the Ghost module is shown in Figure 3. This module is taken from the GhostNetV2 network architecture, which is a lightweight convolutional neural network specifically designed for applications on mobile devices. Its main component is the Ghost module, a novel plug-and-play module. The Ghost module is designed to use fewer parameters to generate more feature maps. The Ghost module consists of three parts, as shown in Figure 3; the first part uses pointwise convolution in depth wise separable convolution, which is a 1 × 1 ordinary convolution. To keep the number of parameters as low as possible, the number of channels was strictly controlled:


(1) $$Y^{\prime }=X*F_{1\times 1}\text{,}$$

In Equation 1, * represents the convolution operation, $F_{1\times 1}$ is the pointwise convolution layer, and Y' is the first part of the output.

The second part is further subdivided into two steps; in the first step, the input feature map is deeply convolved once using a convolutional layer with a kernel size of 3×3 , stride 1, and padding 1. The second step performs a CONCAT operation on the result generated after the first convolution step and the result output from the first part:


(2) $$Y=\mathrm{Concat}\left(\left[Y',Y'*F_{dp}\right]\right)\text{,}$$

In Equation 2, $F_{dp}$ is the depth convolution, and Y is the final output.

The third component is also the main part that distinguishes GhostNetV2 from GhostNet (Han et al., 2020). In GhostNet, although the Ghost module can greatly reduce the computational cost, its feature representation ability is also weakened because “the convolution operation can only model local information within a window.” The spatial information of half of the features is captured by a low-cost operation ( 3×3 depthwise convolution), and the rest of the features are simply obtained by 1×1 pointwise convolution, without any information exchange with other pixels. Therefore, GhostNetV2 additionally introduces the third part to solve the problems such as the lack of feature processing ability of the Ghost module in GhostNet.

**Figure 4:**
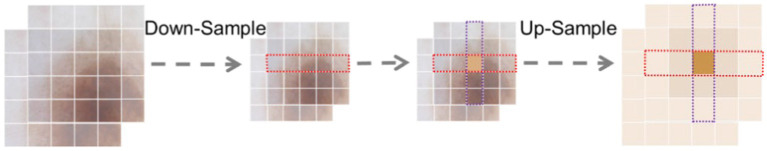
Overview diagram of DFC attention.

The third part is actually a branch of the DFC attention mechanism (in Figure 4), and first avg. pooling performs down-sampling. Then, it passes through 1×1 convolution, followed by horizontal FC and vertical FC. Here, the convolution is used to replace the FC convolution kernel size of the two directions with (1, 5) and (5, 1). Finally, the output of the DFC branch is obtained by sigmoid. The output of the DFC branch is upsampled by bilinear interpolation to obtain the original input size and then multiplied by the output of the original Ghost module to obtain the final output.

The Ghost module makes the network lightweight, and the DFC attention mechanism branch is responsible for the dependence between pixels. Experiments have found that the Ghost module applied to U-Net focuses on a small range of local regions when the number of layers is small. Specifically, some cross-shaped patterns are shown when convolving at lower layers (5 layers), indicating that patches from vertical/horizontal lines are more involved. As the depth (15 layers) increases, the pattern of the attention matrix diffuses. However, it is difficult to reach 15 layers in U-Net, so an ELA attention mechanism was added in the encoder part. The ELA mechanism is also designed to provide long-distance dependencies to the spatial part and enhance the connection between pixels. The dual interaction of the Ghost module and ELA mechanism can play a superposition effect, which is better than a single effect and has a small number of parameters.

### 2.3 ELA module

The ELA mechanism is an improvement over the CA mechanism, and the 2D Conv and BN (batch normalization) layers adopted by the CA mechanism are replaced by the 7×7 1D conv and GN (group normalization) layers, which effectively enhances the interaction and generalization ability of location information embedding so that the entire ELA can accurately find the region of interest.

**Figure 5:**
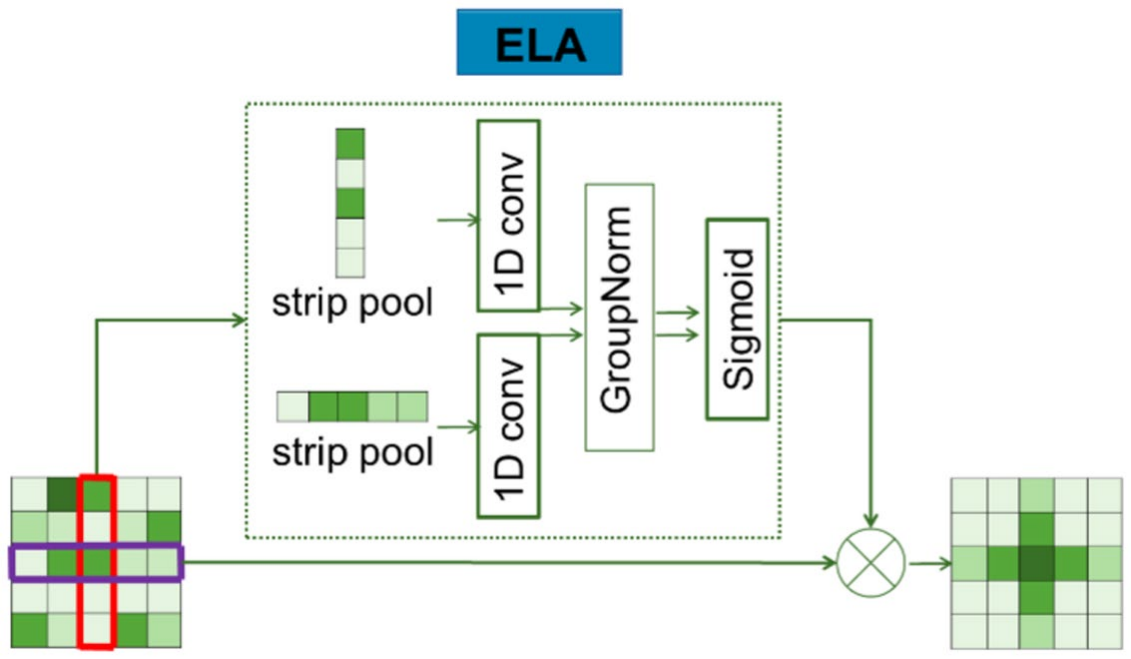
Detailed structure of the ELA.

ELA consists of two main steps: coordinate information embedding and coordinate attention generation. In the first step, long-distance spatial dependencies are captured by using strip pooling instead of spatial global pooling. The specific process is shown in Figure 5.

Average pooling was firstly performed on the features of the input H X W for each channel in two spatial scales: (H,1) horizontally and (1,W) vertically. Using strip pooling (pooling only on the dimensions of H or W), the mathematical formula for the features of H×1 and 1×W as follows:


(3) $$z_{c}^{h}\left(h\right)=\frac{1}{H}\underset{0\leq i<H}{\sum }x_{c}\left(h,i\right)\text{,}$$

(4) $$z_{c}^{w}\left(w\right)=\frac{1}{W}\underset{0\leq j<W}{\sum }x_{c}\left(j,w\right)\text{,}$$

$z_{c}^{h}$ is the output representation of the C-th channel at height h, and $z_{c}^{w}$ is the output representation of the C-th channel at width w.


(5) $$y^{h}=\sigma \left(G_{n}\left(F_{h}\left(z_{h}\right)\right)\right)\text{,}$$

(6) $$y^{w}=\sigma \left(G_{n}\left(F_{w}\left(z_{w}\right)\right)\right)\text{,}$$

The localization information obtained from Equations 3, 4 is then embedded, and a novel encoding method is used to generate an accurate location attention map. The detailed description of the process is as follows: $z_{h}$ and $z_{w}$ , obtained by Equations 3, 4, both capture the global sensory field and contain precise location information. In order to effectively utilize these features, the conv2D feature processing mode is overruled, as it is usually more appropriate to use 1D convolution instead of 2D convolution for processing these sequential signals: 1D convolutions are not only good at processing sequential signals but also more lightweight than 2D convolutions; therefore, 1D convolutions are applied to enhance the position information in horizontal and vertical directions. Subsequently, the GN layer was used to process the enhanced location information, resulting in the location attention representation in the horizontal and vertical directions, as described in Equations 5, 6.

In the above description, σ denotes the nonlinear activation function, $F_{h}$ and $F_{w}$ are used to denote one-dimensional convolution, and the convolution kernel size of $F_{h}$ and $F_{w}$ is set to 7. The position attention representations in the horizontal and vertical directions are denoted by $y^{h}$ and $y^{w}$ , respectively. Finally, the output of ELA is obtained through Equation 7, and the result is denoted as Y.

The existence of the Ghost module and ELA mechanism mainly provides long-distance dependence; thus, in the up-sampling stage, it introduced the attention gate in Attention U-Net to enhance the spatial local attention mechanism in order to reduce the amount of parameters while ensuring the effect. This makes the network pay more attention to the region of interest and analyze the edge features of medical images more clearly.


(7) $$Y=x_{c}\times y^{h}\times y^{w}\text{,}$$

### 2.4 Attention gate

**Figure 6:**
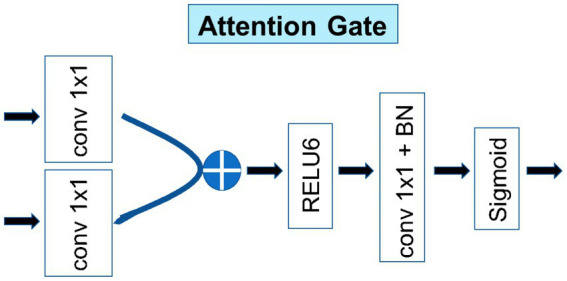
Detailed structure of the attention gate.

As shown in Figure 6, the other main purpose of applying an attention block to the decoder architecture is to improve the local (ROI) features and suppress some non-interest regions. Taking the output of the previous layer as input, it passed the feature maps with the same number of channels through A convolution with kernel 1 and outputted two feature maps, A and B. Then, the A and B feature maps (provided that the A and B feature maps were the same size) were added to obtain the C feature map.

**Figure 7:**
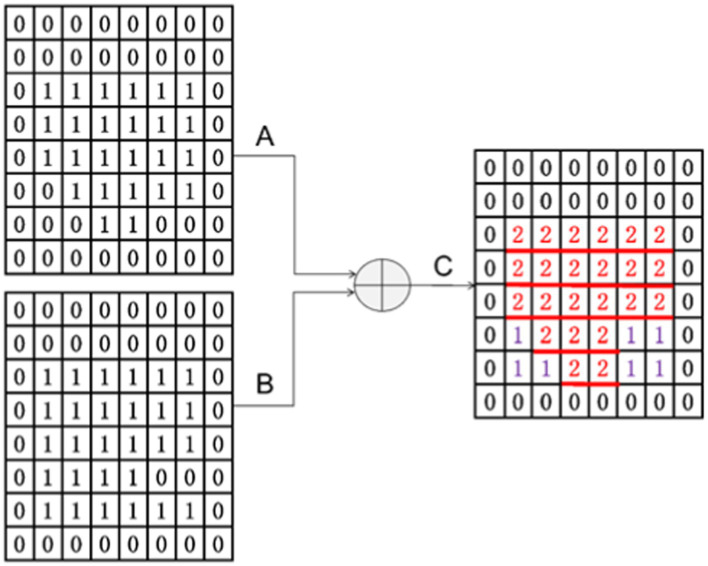
Results of attention gate convolution.

From Figure 7, it can see why the size of A and B should be the same after convolution. They can only be added when they are the same size. From C, it can see that the attention gate has a primary effect of increasing the local attention value. A + B essentially reinforces the signal value of the same region of interest, which is the red part in the figure. The regions that are different from A and B are also included as auxiliary functions, and their effects cannot be ignored. These effects are denoted in purple in the figure.

**Figure 8:**
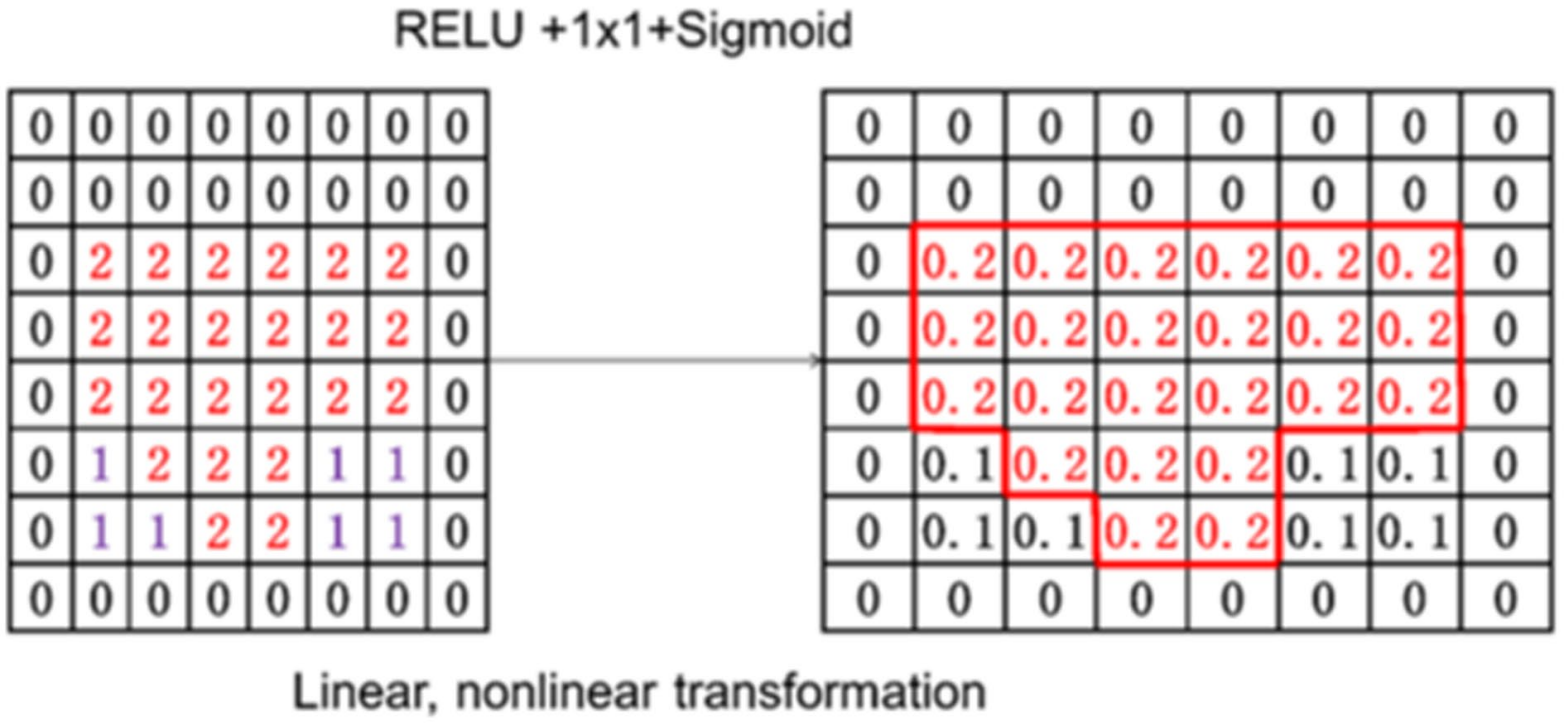
Attention weight transformation diagram.

As shown in Figure 8, the output C feature map was transformed linearly and non-linearly, and a value for each patch was provided by using a sigmoid function. This value is the attention weight for the next step.

## 3 Results and discussion

In this section, comprehensive experiments were conducted on MSGU-Net for the skin lesion segmentation task. Specifically, the performance of MSGU-Net on the medical image segmentation task on ISIC2017 and ISIC2018 was evaluated.

### 3.1 Implementation details

The image size datasets in ISIC2017 and ISIC2018 were resized to 256×256 according to previous work. To prevent overfitting, data augmentation techniques such as random flipping, random rotation, and others were used. The BCEWithLogitsLoss was used for ISIC2017 and ISIC2018 datasets. The batch size was set to eight and the Adam optimizer was used with an initial learning rate of 1e-4. ReduceLROnPlateau was used as the scheduler, with a minimum learning rate of 1e-5. The training period was set to 100. MSGU-Net does not use pretrained weights, and all models appearing above are randomly initialized. All the experiments were performed on a single NVIDIA GeForce RTX 2060 GPU.

Since the experimental dataset is used for binary classification, the loss function of multi-classification is not adopted. Binary cross entropy (BCE), which does not perform any processing, measures the binary cross entropy function between the output and the target, and the binary cross entropy is set at [0.1]. Meanwhile, MSGU-Net uses the BCEWithLogitsLoss, which integrates the sigmoid layer with the BCELoss, which is more stable than simply adding the sigmoid layer with the BCELoss because the LogSumExp trick is used to obtain numerical stability.

### 3.2 Comparison on lesion boundary segmentation challenge

In order to further verify the effectiveness of the proposed network MSGU-Net, it is comprehensively compared with the typical methods in the field of medical image segmentation in recent years. At the same time, in order to ensure the diversity of the network, U-Net (Ronneberger et al., 2015), UNet++, SegNet (Badrinarayanan et al., 2017), FAT-Net (Wu et al., 2022), UNeXT-L (Valanarasu and Patel, 2022), Swin-Unet (Cao et al., 2023), and TransUNet (Chen et al., 2021) are selected for the comparison network of the ISIC2017 dataset. For the comparison network of the ISIC2018 dataset, U-Net (Ronneberger et al., 2015), UNet++, Attention U-Net (Jensen et al., 2018), TransFuse (Zhang et al., 2021), UTNetV2 (Gao et al., 2022), and SANet (Hu et al., 2021) are selected.

The U-Net network framework is composed of an encoder and a decoder. In each layer of the encoder, maximum pooling is utilized to downscale the feature map dimensions and simultaneously double the number of channels. Conversely, in each layer of the decoder, bilinear interpolation is employed to upscale the feature map, thereby reducing the channel count and effectively restoring the dimensions of the lesion area. UNet++ reduces the semantic gap between encoder and decoder by using a series of nested and dense skip connections. The SegNet network structure aims to accurately extract pixel-level semantic information from images and accurately segment medical images. FAT-Net combines the feature pyramid network and attention mechanism to improve the performance of segmentation. Swin-UNet, TransUNet, and TransFuse improve the network’s ability to model global context information by introducing transformers into convolutional networks. UNeXT-L improves the attention mechanism to improve the efficiency of the network modeling long-term dependencies. Attention U-Net adds an attention module to U-Net, which is used to dynamically adjust the attention of the network to different regions to improve the performance and accuracy of the network. UTNetV2 separates semantic information from high-level and low-level features by using finder details of Hadamard multiplication captured in the features; these are integrated into each level of the feature map generated by the encoder. SANet improves the visual attention module (the SE module) to obtain the SA module, which captures global and local context information at the same time and constructs SANet to complete the semantic segmentation task. The above diversity network was experimented with in the same experimental environment, and the comparison results are shown in Table 1, where the test optimal performance index is indicated by bold.

**Table 1:** Performance comparison table between MSGU-Net and other networks.

Data	Model	Params	GFLOPs	mIoU	DSC
	U-Net	31.13	55.84	73.61	82.53
	UNet++	9.16	34.65	75.30	84.01
	SegNet	17.94	22.35	69.63	82.14
ISIC2017	FAT-Net	28.23	42.83	76.52	85.05
	UNeXt-L	3.80	**1.08**	75.43	84.02
	Swin-UNet	25.86	5.86	76.71	85.03
	TransUNet	105.32	38.52	**77.53**	84.74
	**MSGU-Net(ours)**	**1.22**	**4.12**	**77.03**	**85.53**
	U-Net	31.13	55.84	74.55	84.03
	UNet++	9.16	34.65	76.12	84.96
	Attention U-Net	8.73	16.74	76.63	86.11
ISIC2018	TransUNet	105.32	38.52	80.51	88.91
	TransFuse	26.27	11.53	80.63	**89.27**
	UTNetV2	12.80	15.50	78.97	88.25
	SANet	23.90	5.99	79.52	88.59
	**MSGU-Net(ours)**	**1.22**	**4.12**	**81.40**	**88.74**

Bold values represent the emphasis on key data.

Table 1 presents a comparative analysis of the performance metrics, number of parameters (Params) and computational complexity (GFLOPs) for the MSGU-Net network and other networks on the ISIC2017 and ISIC2018 datasets. The experimental results show that MSGU-Net not only outperforms its counterparts but also demonstrates significant advantages in terms of computational complexity and the number of parameters required. Through a rough analysis of the evaluation indicators of the ISIC2017 dataset, it is found that compared with the transformer-based network, the network parameters of MSGU-Net are 4.7% of that of Swin-UNet and 1.2% of that of TransUNet, and the computational complexity of MSGU-Net is significantly reduced, which shows that MSGU-Net is an effective improvement. Compared with the base CNN network (U-Net), MSGU-Net has obvious advantages in terms of segmentation performance, number of parameters and computational complexity, indicating that MSGU-Net has good segmentation results. The Dice-Sørensen coefficient (DSC) of the MSGU-Net network for the ISIC2017 dataset has reached the leading level among many networks. A detailed analysis of the evaluation metrics of the ISIC2018 dataset shows that MSGU-Net has obvious advantages over other improved CNN-type networks: the DSC co-efficient is 4.71% higher than that of U-Net, 3.78% higher than that of UNet++, and 2.63% higher than that of Attention U-Net, which also uses an attention gate in the up-sampling stage. Compared to transformer-based networks, MSGU-Net is able to match or even sur-pass the performance of TransUNet and TransFuse with a miniscule number of parameters.

**Figure 9:**
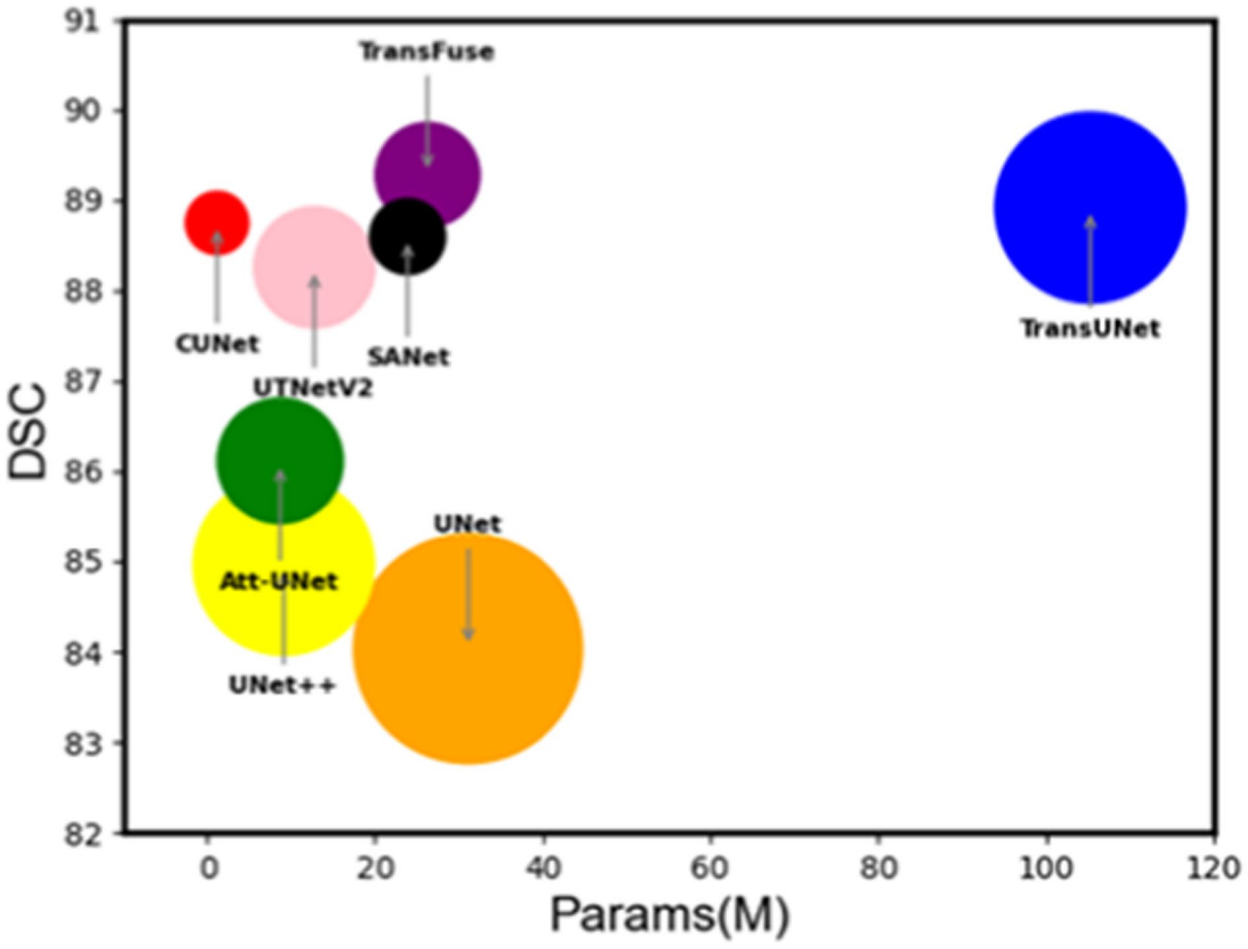
Bubble plots of individual network performance on the ISIC2018 dataset.

In Figure 9, the DSC coefficient and Params (M) are plotted according to the effect of each network on the ISIC2018 dataset, and the size of the bubble circle in the figure is measured more by the GFLOPs (Giga floating point operations per second) of each network. It is easy to see that MSGU-Net and the family of networks that introduce trans-formers to convolutional networks—TransFuse and TransUNet— are the best algorithms. However, MSGU-Net is significantly better than other network frameworks in terms of computational complexity and the number of parameters, which are important characteristics to consider when developing applications.

Embedded devices not only have certain requirements for the number of parameters and calculation, but also have strict requirements for the segmentation performance. The following is the segmentation results of different networks on the ISIC2017 and ISIC2018 datasets. From left to right are the segmentation results of original image, Ground Truth (GT), UNet, UNet ++, TransUNet, TransFuse, MedT, UNeXt, and MSGU-Net.

**Figure 10:**
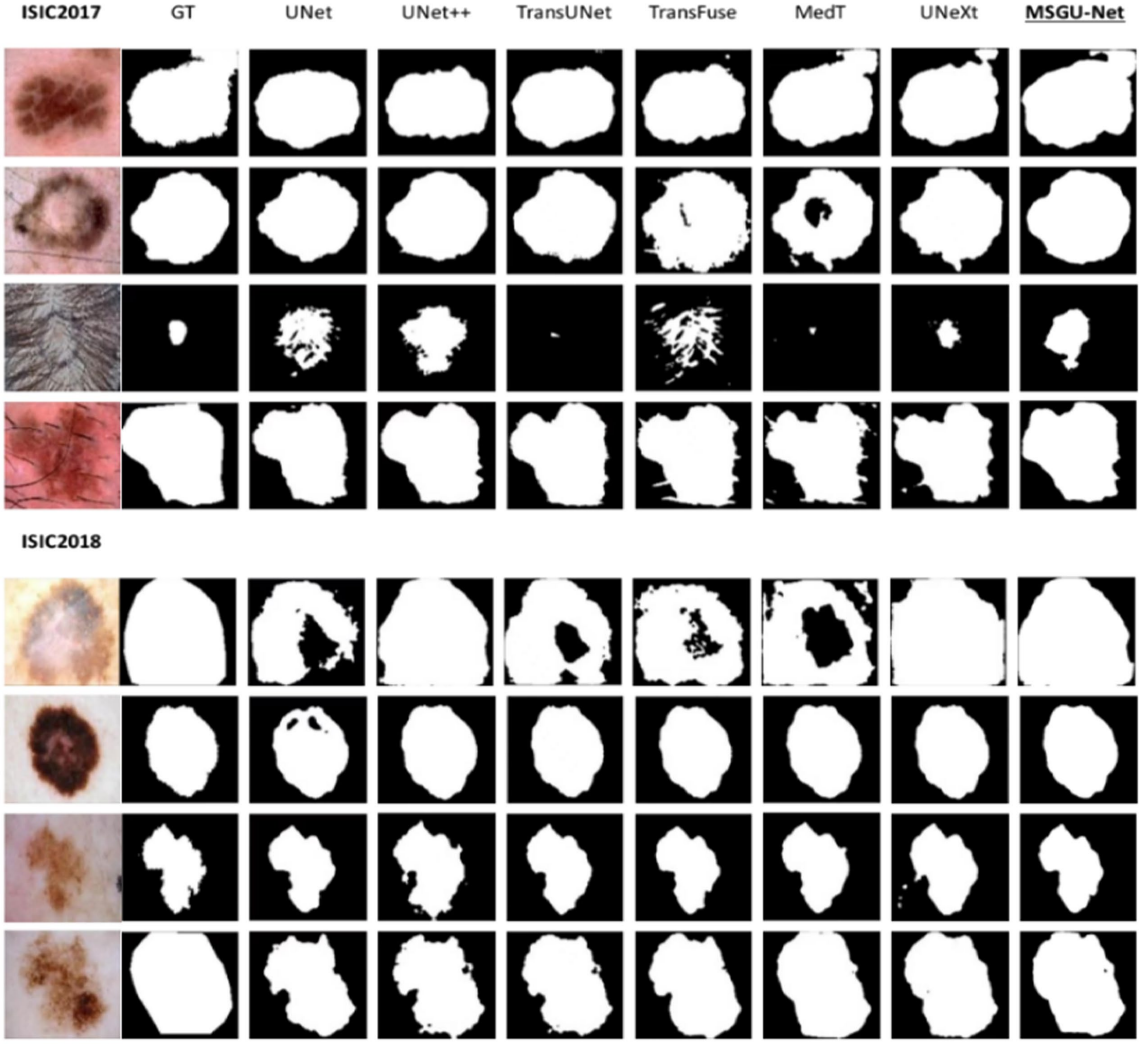
Comparison of segmentation results.

From the segmentation results shown in Figure 10, it is clear that MSGU-Net and the introduction of the transformer into the convolutional networks TransFuse and TransUNet demonstrate great advantage in dealing with image boundary details. In the four-image segmentation of ISIC2017, MSGU-Net processed the first, second, and fourth images effectively, showing the ability to surpass most models in both content presentation and detail processing. However, due to the influence of hair in the third image, the processing of this image presented problems of varying degrees for each model. In four images from ISIC2018, MSGU-Net presents segmented skin images with near-perfect performance. In summary, MSGU-Net is able to deal with most skin images very well, partly because excessive hair occlusion will affect the segmentation effect, but it is still able to effectively segment the images with hair occlusion.

### 3.3 Ablation experiment

To understand the role of each module implemented in the network, separate ablation experiments were also conducted on the ISIC2018 dataset. In Table 2, from top to bottom, MSGU-Net is composed of the SPP-Inception module, Ghost module, and ELA attention mechanism module, and I G C is composed of the SPP-Inception module, Ghost module, and CBAM attention mechanism module. C G E and C G C are the SPP-Inception module replaced by the regular convolution module, the Xception in X G E and X G C is the ap-plication of MobileNet’s depth wise separable convolution to the Inception module, and D G E and D G C are the superposition of the two-layer Ghost module. D I E and D I C are the superposition of two layers of SPP-Inception modules. In order to more accurately judge the role of the module, here are three additional performance metrics: accuracy (ACC), sensitivity (Sen), and specificity (Spe). ACC is the ratio of the number of samples correctly predicted by the classification model to the total number of samples, that is, the number of correctly classified samples divided by the total number of samples. The higher the accuracy, the better the prediction effect of the model as a whole. Sen is referred to as true positive rate (TPR) and is the fraction of all examples that are actually positive that the model successfully predicts to be positive. Sensitivity measures the ability of the model to identify positive examples, that is, the accuracy of the model to predict positive examples. Spe is the fraction of all samples that are actually negative that the model successfully predicts as negative. Specificity measures the ability of the model to identify negative examples, that is, the prediction accuracy of the model for negative examples.

**Table 2:** Performance comparison table between MSGU-Net and other networks.

Net	Conv	Inc	D-Inc	Xce	Ghost	D-Ghost	ELA	CBAM	mIoU	DSC	Acc	Spe	Sen
MSGU- Net(ours)	□	☑	□	□	☑	□	☑	□	**81.40**	**88.74**	**94.86**	**97.13**	**90.05**
I G C	□	☑	□	□	☑	□	□	☑	81.02	88.34	94.79	96.91	90.01
C G E	☑	□	□	□	☑	□	☑	□	78.97	87.05	93.25	95.84	88.60
C G C	☑	□	□	□	☑	□	□	☑	78.43	86.53	93.00	96.92	86.85
X G E	□	□	□	☑	☑	□	☑	□	79.16	87.29	93.38	95.53	89.34
X G C	□	□	□	☑	☑	□	□	☑ ☑	78.76	86.86	93.17	96,47	88.16
D G E	□	□	□	□	□	☑	☑	□	79.15	87.22	93.47	95.85	89.47
D G C	□	□	□	□	□	☑	□	☑	78.99	87.01	93.31	95.61	89.17
D I E	□	□	☑	□	□	□	☑	□	78.75	86.83	93.21	95.10	89.42
D I C	□	□	☑	□	□	□	□	☑	78.46	86.52	93.10	95.13	89.00

By comparing MSGU-Net (I G E) and I G C, C G E and C G C, X G E and X G C, D G E and D G C, and D I E and D I C, it can be concluded that the performance improvement brought by the ELA attention mechanism far exceeds that of the CBAM attention mechanism. Through the comparative analysis of MSGU-Net (I G E), C G E, X G E, D G E, and D I E, it can be concluded that the multi-scale feature extraction of the SPP-Inception module is very effective. The DSC brought by CGE conventional convolution is 87.05, and the DSC brought by the MSGU-Net (I G E) SPP-Inception module is 88.74. Obviously, the SPP-Inception module brings more powerful feature extraction capabilities. By comparing the DSC results of MSGU-Net (I G E) and D I E, it can be concluded that using only the SPP-Inception module is not enough because the SPP-Inception module cannot provide long-distance dependencies. The comparison of DSC results between MSGU-Net (I G E) and D G E shows that it is also not enough to use the Ghost module only for feature ex-traction and to provide long-distance dependencies. Compared with the multi-scale feature extraction and fusion of SPP-Inception, the feature extraction ability of the Ghost module is weak. Finally, it was determined that the Inception module and Ghost module should cooperate with each other to enhance multi-scale feature extraction and provide long-distance dependence; thus producing the MSGU-Net network architecture.

**Figure 11:**
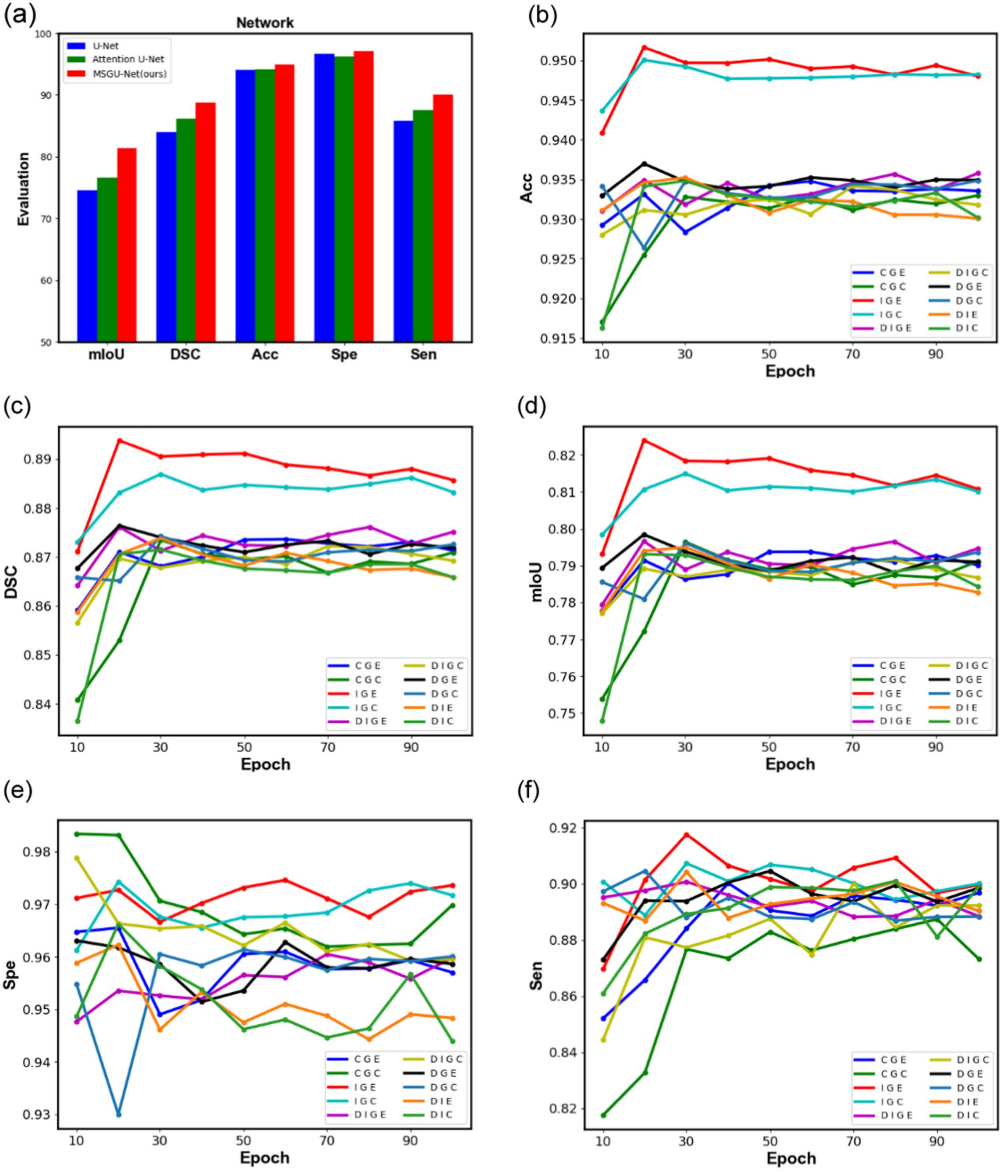
Comparison graphs of different module combination networks in ISIC2018 dataset, **(A)** is a bar chart comparing the performance of the baseline network U-Net, Attention U-Net, and MSGU-Net, **(B–F)** represent the performance graphs of different module combination networks, respectively.

From Figure 11A, it can clearly be seen that the indicators of all aspects of the improved network have been significantly increased. MSGU-Net surpassed the baseline network, U-Net, in all aspects, with mIoU increased by 6.85% and DSC increased by 4.71%. Moreover, MSGU-Net out-performs.

Attention U-Net which also uses an attention gate in the up-sampling stage, with an increase of 4.77% in mIoU and 2.63% in DSC. These data are enough to prove the effectiveness of our improvement and can provide promotional information for the proposed network.

To further observe the role of different modules in the network, the changes of their evaluation metrics on the ISIC2018 test set during the training process were visualized, and the curves are shown in Figure 11. From Figures 11B–D, it can be clearly seen that the combination of the SPP-Inception module and Ghost module is far ahead of other combinations in the three indicators DSC, ACC and mIoU, and the DSC coefficient is as high as 89.37%, which means that they can better process the features of the image. The proposed method can effectively segment the image mask correctly. From Figures 11B–D, it can also clearly be seen that the positive effects of long-distance dependencies provided by the ELA attention mechanism: identifying and segmenting different structures in the image more accurately and handling noise and artifacts in the image, thus improving the accuracy of the segmentation results. The two figures in Figures 11E,F show that, compared with other combinations, the combination of the SPP-Inception module and Ghost module can identify regions of interest (such as lesions, organs, etc.) and distinguish non-interest regions (such as background, normal tissue, etc.) with more stability.

## 4 Conclusions and future works

In this paper, two advanced modules are introduced. The synergy between the SPP-Inception module and the ghost module integrates low-level and high-level features, achieving multi-scale information fusion within a pyramid structure. An efficient local attention (ELA) mechanism and an attention gate mechanism are employed to precisely identify the region of interest (ROI). Leveraging these modules and mechanisms, the MSGU-Net is developed for skin lesion segmentation tasks. The experimental results indicate that MSGU-Net can function as a lightweight deep neural network, suitable for deployment across various intelligent devices and mobile platforms, showing significant potential for broad adoption.

U-Net is a deep learning model originally designed to solve medical image segmentation problems. MSGU-Net, as a variant of it, can also achieve excellent results in the field of medical images, especially in tasks such as cell segmentation, organ localization and pathological image analysis. The structural characteristics of MSGU-Net make it perform better in processing small objects and details in images, which is particularly important in medical image analysis. Of course, the application of U-Net is not limited to medical images, it is also excellent in remote sensing image processing, autonomous driving technology, industrial inspection and other fields. The MSGU-Net of this paper, as mentioned above, has great potential in these aspects. Again, this paper focuses on significantly reducing the number of parameters and computational complexity while improving performance, and further research on mobile deployment and other applications of MSGU-Net will be done in the future.

At present, semi-supervised learning and self-supervised learning have already emerged as one of the mainstream research directions within the domain of medical images. They are committed to addressing the problem of limited labeled data, which differs to some degree from our main focus on optimizing the network architecture itself. Nevertheless, their approaches can act as valuable references in those situations where obtaining annotated data turns out to be extremely difficult. Additionally, there is a significant amount of work that aims to handle memory constraints when dealing with large biomedical images. Although our MSGU-Net is not specifically designed for processing large images, it might potentially derive benefits from the concepts and principles underlying such efforts. Considering the limitations of the algorithm as well as future research directions, on the one hand, this paper underlines the necessity of considerably reducing the number of parameters and computational complexity while also enhancing performance. As a result, we plan to deploy MSGU-Net in a real-world setting in our upcoming work. On the other hand, currently, MSGU-Net is solely tailored for skin lesion segmentation tasks. Hence, in the future, we aim to extend its lightweight design to other tasks.

## Data Availability

The original contributions presented in the study are included in the article/supplementary material, further inquiries can be directed to the corresponding author.
